# Retraction of: Exosomal miR-200c suppresses chemoresistance of docetaxel in tongue squamous cell carcinoma by suppressing TUBB3 and PPP2R1B

**DOI:** 10.18632/aging.206148

**Published:** 2024-10-31

**Authors:** Jun Cui, Haiyan Wang, Xiaohe Zhang, Xiaodong Sun, Jin Zhang, Jinji Ma

**Affiliations:** 1 Department of Dental Implantology, Jinan Stomatological Hospital, Jinan 250001, Shandong Province, China; 2 Department of Ultrasound, Shandong Provincial Qianfoshan Hospital, The First Hospital Affiliated with Shandong First Medical University, Jinan 250014, Shandong Province, China; 3 Department of Oral Disease Gaoxin Branch, Jinan Stomatological Hospital, Jinan 250001, Shandong Province, China

**Keywords:** tongue squamous cell carcinoma, docetaxel, chemoresistance, miR-200c, TUBB3, PPP2R1B

**This article has been retracted:** Aging has completed its investigation of this paper. We found several instances of overlap between figures and replication of tumor images from a previously published unrelated paper. Specifically, two transwell assay images in Figure 6C, representing cell migration data, overlap panels 1 and 3, and panel 5 overlaps panel 1 in Figure 3H, illustrating data from invasion assays. Panel 3 in Figure 6B, representing data from invasion assays, overlaps panel 4, while panel 1 in Figure 6B overlaps panel 5 in Figure 8K, illustrating cell migration ability. In addition, transwell assay images in Figures 1E and F overlap images from an unrelated, recently retracted paper [1]. We found that tumor images in Figure 7E replicate tumor images in Figure 4F of a paper published earlier by a different group of authors [2]. Moreover, the same images were later reproduced in [3], which also used tumor images in Figure 4E. Additionally, the same distinctively scratched ruler was used to measure xenograft tumors in a number of other papers from unrelated teams of authors [2–4].

The Scientific Integrity office at Aging contacted the authors, but they did not respond to requests to clarify, and the paper was retracted based on an Editorial decision. The Scientific Integrity office also notified the authors’ Institutions about this retraction and added their names to an Editorial Warning list.
